# The Prognostic Value of a Four-Dimensional CT Angiography-Based Collateral Grading Scale for Reperfusion Therapy in Acute Ischemic Stroke Patients

**DOI:** 10.1371/journal.pone.0160502

**Published:** 2016-08-09

**Authors:** Sheng Zhang, Weili Chen, Huan Tang, Quan Han, Shenqiang Yan, Xiaocheng Zhang, Qingmeng Chen, Mark Parsons, Shaoshi Wang, Min Lou

**Affiliations:** Department of Neurology, the Second Affiliated Hospital of Zhejiang University, School of Medicine, Hangzhou, China; Medizinische Universitat Innsbruck, AUSTRIA

## Abstract

**Objective:**

Leptomeningeal collaterals, which affects tissue fate, are still challenging to assess. Four-dimensional CT angiography (4D CTA) originated from CT perfusion (CTP) provides the possibility of non-invasive and time-resolved assessment of leptomeningeal collateral flow. We sought to develop a comprehensive rating system to integrate the speed and extent of collateral flow on 4D CTA, and investigate its prognostic value for reperfusion therapy in acute ischemic stroke (AIS) patients.

**Methods:**

We retrospectively studied 80 patients with M1 ± internal carotid artery (ICA) occlusion who had baseline CTP before intravenous thrombolysis. The velocity and extent of collaterals were evaluated by regional leptomeningeal collateral score on peak phase (rLMC-P) and temporally fused intensity projections (tMIP) (rLMC-M) on 4D CTA, respectively. The cutoffs of rLMC-P and rLMC-M score for predicting good outcome (mRS score ≤ 2) were integrated to develop the collateral grading scale (CGS) (rating from 0–2).

**Results:**

The CGS score was correlated with 3-months mRS score (non-recanalizers: *ρ* = -0.495, *p* = 0.01; recanalizers: *ρ* = -0.671, *p* < 0.001). Patients with intermediate or good collaterals (CGS score of 1 and 2) who recanalized were more likely to have good outcome than those without recanalization (*p* = 0.038, *p* = 0.018), while there was no significant difference in outcome in patients with poor collaterals (CGS score of 0) stratified by recanalization (*p* = 0.227).

**Conclusions:**

Identification of collaterals based on CGS may help to select good responders to reperfusion therapy in patients with large artery occlusion.

## Introduction

Leptomeningeal collaterals (LMCs) are important to maintain the survival of ischemic brain tissue by compensating the decreasing cerebral blood flow, in the setting of acute cerebral artery occlusion [1]. Many studies have interpreted it as a radiologic surrogate to predict the response of revascularization therapy [2,3]. Although digital subtraction angiography (DSA) is the gold standard to visualize the LMCs, it has the disadvantages of an invasive technique. Conventional CT angiography (CTA) can provide noninvasive methods to assess the distribution of LMCs rapidly. A post-hoc analysis on Multicenter Randomized Clinical Trial of Endovascular Treatment of Acute Ischemic Stroke in the Netherlands (MR CLEAN) data has proved that collateral status based on conventional CTA modified the treatment effect of endovascular therapy [4]. However, the inter-rater agreement of collateral status on conventional CTA was only moderate (κ = 0.60) in that study. Moreover, conventional CTA failed to acquire dynamic information [5]. Although the other study has found that multiphase CTA which provides time-resolved images of leptomeningeal collateral vessels was useful in selecting patients who may benefit from endovascular therapy, it excluded patients with absent or poor collaterals, whereas it remains unproven whether patients without fulfilling neuroimaging inclusion criteria based on multiphase CTA would achieve little treatment effect [6]. Compared with multiphase CTA, four-dimensional (4D) CTA, derived from CT perfusion (CT), is also noninvasive and better estimates collateral status because it captures the whole passage process of contrast bolus [7–9]. It is thus reasonable to use 4D CTA to simulate the manner used on DSA by grading both the speed and extent of LMCs, so as to assess whether collateral status is able to modify the effect of reperfusion therapy in real world.

Most recently, LMCs assessed on 4D CTA, with either peak phase or temporally fused maximum intensity projection (tMIP), have been found to be associated with clinical outcome [10]. The tMIP captures the entire transit of the contrast bolus, which could reveal the extent of LMCs distribution, while at peak phase of the time attenuation curve, comparison of vascularity between the unaffected and ischemic hemispheres reflects the speed of collateral filling [8]. Therefore, in this study, we proposed a novel method integrating information from both peak phase and tMIP on 4D CTA to grade velocity and extent of collaterals together, similar to DSA grading. We then investigated whether this novel method could identify patients who were likely to benefit from reperfusion therapy.

## Methods

### Ethics Statement

Written informed consent was obtained from each patient or an appropriate family member. The human ethics committee of The Second Affiliated Hospital of Zhejiang University approved the protocol of this study. All clinical investigations were conducted according to the principles expressed in the Declaration of Helsinki.

### Patients selection

We retrospectively reviewed our prospectively collected database for acute ischemic stroke (AIS) patients who were admitted to our center between June 2011 and April 2015. Patients were included if: i) received intravenous thrombolysis (IVT) within 6 hours after onset; ii) with middle cerebral artery (MCA) M1 segment and / or internal carotid artery (ICA) occlusion; iii) underwent CTP at baseline, and noncontrast CT (NCCT) or diffusion-weighted imaging (DWI) at 24 hours after IVT; iv) had hypoperfusion at baseline CTP with corresponding symptoms; v) had follow-up modified Rankin scale (mRS) score at 3 months; vi) prestroke mRS ≤ 2. Patients were excluded if: i) antegrade flow across incomplete occlusion; ii) severe motion artifacts. Intravenous rt-PA was administered according to the international guidelines (0.9mg/kg, 90mg dose at maximum, 10% in a bolus in 1 minute with the remaining dose in a 60-min infusion). This study was approved by the human research ethics committee of the Second Affiliated hospital of Zhejiang University.

### Imaging protocol

Baseline CTP was performed on a 64-slice CT scanner (SOMATZOM Definition Flash; Siemens Healthcare Sector, Forchheim, Germany) before initiation of IVT, including non-enhanced head CT scan (120 kV, 320 mA, contiguous 5 mm axial slices, acquisition time: 7 seconds), and volume perfusion CT (VPCT) (100 mm in the z-axis, 4 seconds delay after start of contrast medium injection, 74.5 seconds total imaging duration, 80 kV, 120 mA, slice thickness 1.5 mm, collimation 32×1.2 mm). VPCT consisted of 26 consecutive spiral acquisitions of the brain. A 60-mL bolus of contrast medium (Iopamidol; Braccosine, Shanghai, China) was used at a flow rate of 6 ml/s, followed by a 20 mL saline chaser at 6 ml/s. The effective dose (calculated by multiplying dose-length products with published conversion factors) was 3.68 mSV for VPCT and 2.19 mSV for NCCT acquisition.

### Assessment of collaterals on 4D CTA

All baseline and follow-up images were reviewed and reconstructed using commercial software (MIStar; Apollo Medical Imaging Technology, Melbourne, Australia). 4D CTA was reconstructed from baseline VPCT. Prior to the reconstruction, baseline VPCT source images were reviewed (5 seconds per frame via a movie player tool of MIStar) by 1 stroke fellow (S.Z, with over 3 years of experience in stroke imaging) in order to confirm the contrast visualized beyond the complete occlusion was truly from retrograde filling. Patients with antegrade flow were excluded from this study during this review process.

As described by Frölich et al, baseline 4D CTA images were reconstructed from VPCT in axial, coronal and sagittal with 20-mm-thick slabs [8,9]. Peak phase was defined as the point of peak arterial opacification identified on the time attenuation curve in the unaffected MCA. Temporally fused MIP (tMIP) reconstruction that fused contrast opacification across the entire duration of the 4D CTA were also obtained.

The regional leptomeningeal collateral (rLMC) score were then graded on peak phase (rLMC-P) and temporally fused maximum intensity projections (tMIP) (rLMC-M) on 4D CTA, respectively, as previously described [5]. S.Z and W.C who were blinded to other imaging and clinical data independently assessed rLMC score. Intra-class correlation coefficient (ICC) for inter-rater agreement was substantial for rLMC-P (ICC 0.85, 95% CI, 0.74–0.92) and rLMC-M (ICC 0.87, 95%CI, 0.77–0.93), respectively. Cases with over 2 points difference were regraded by consensus.

### Development of integrated collateral grading scale (CGS)

**Fig 1:**
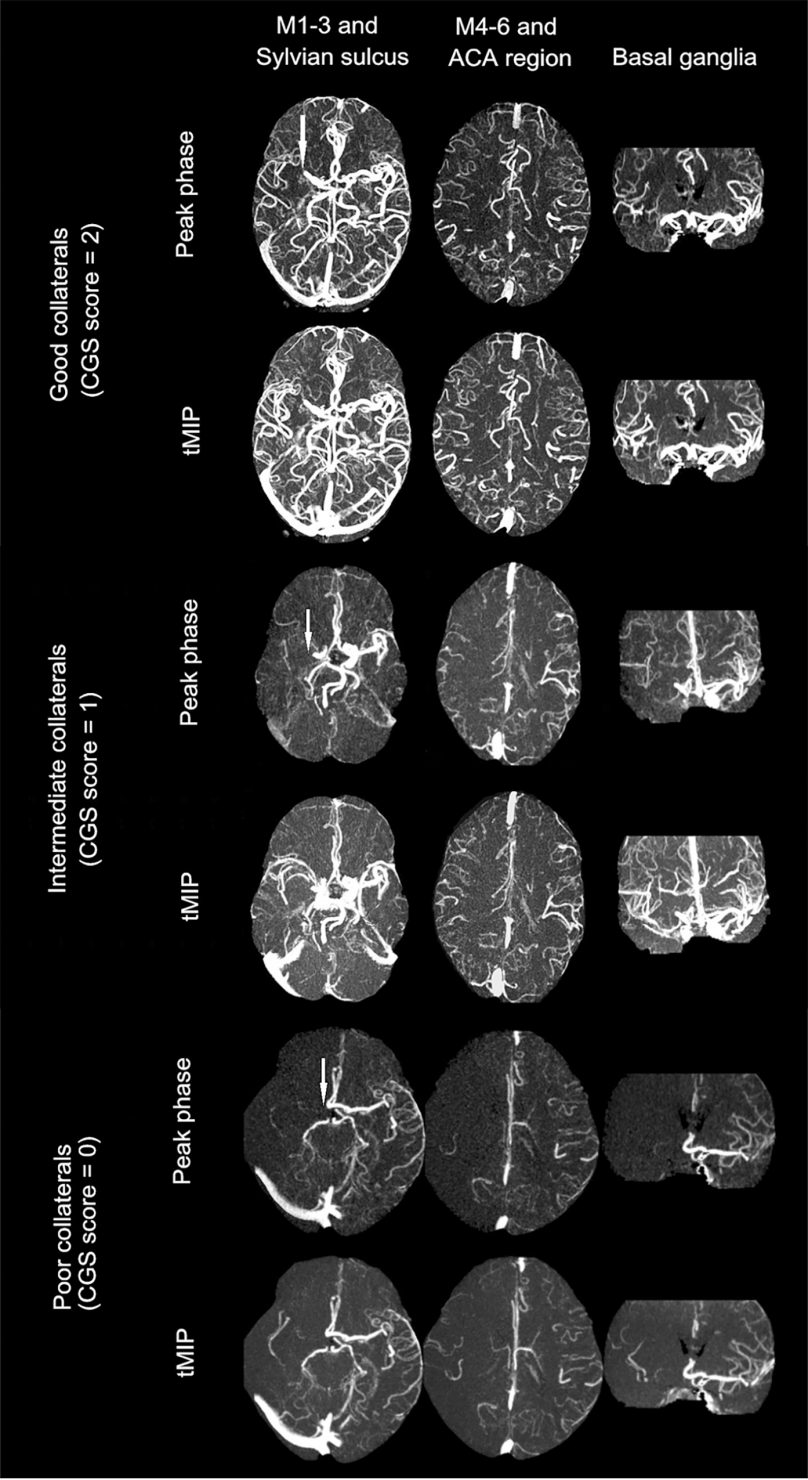
Collateral status on dynamic 4D CTA as measured by collateral grading scale (CGS). White arrow indicates the site of occlusion. Top two rows: a patient with a right MCA M1 segment occlusion and good collaterals as CGS score of 2: rLMC score on peak phase (rLMC-P) = 15, and rLMC score on tMIP (rLMC-M) = 18. Middle two rows: a patient with a right ICA occlusion and intermediate collaterals as CGS score of 1 (rLMC-P = 13 and rLMC-M = 15). Bottom two rows: a patient with a right ICA occlusion and poor collaterals as CGS score of 0 (rLMC-P = 3 and rLMC-M = 4).

Receiver operating characteristic (ROC) curve analysis were used to determine the cutoffs of each rLMC score for predicting good outcome (mRS score ≤ 2). The optimal threshold for rLMC-P was 11.5 (sensitivity, 83.3%; specificity, 66.1%; Youden index, 49.4%), and 16.5 for rLMC-M (sensitivity, 54.2%; specificity, 83.4%; Youden index, 38.1%). Consecutively, an integrated collateral grading scale (CGS) was then developed based on the above thresholds, assigning patients to poor collaterals (score 0: rLMC-P ≤ 11 and rLMC-M ≤ 16), intermediate collaterals (score 1: rLMC-P ≤ 11 and rLMC-M>16, or rLMC-P>11 and rLMC-M ≤ 16), and good collaterals (score 2: rLMC-P >11 and rLMC-M>16) (Fig 1).

### Radiologic and clinical assessment

Thresholds of Tmax > 6 seconds was used for volumetric measurement of baseline and 24-hours hypoperfusion area.[10] Relative cerebral blood flow (rCBF) < 30% was used for calculating baseline infarct volume, and 24-hours DWI or NCCT for the final infarct volume. [11] DWI lesions were assessed with a b value of 1000 s/mm^2^. Volumetric analysis was performed using MIStar software. The baseline mismatch ratio was defined as the volume of Tmax >6 seconds divided by the corresponding rCBF < 30%.[12] Recanalization was assessed based on Arterial Occlusive Lesion (AOL) scale which was rated on 24-hours CTA/MRA by the consensus of 2 stroke fellows (QM, C and SQ. Y). A score of 2 and 3 was classified as recanalization.[13] National Institutes of Health Stroke Scale (NIHSS) at baseline, 24 hours, 7 days, times from onset to imaging, treatment and mRS at 90 days were collected. Patients were dichotomized into good (mRS ≤ 2) versus poor outcome (mRS > 2) at 90 days. Symptomatic hemorrhagic transformation (sHT) was defined as any intracranial hemorrhage associated with an increase of ≥ 4 points on NIHSS, or one leading to death.[14]

### Statistical analysis

All metric and normally distributed variables were reported as mean ± standard deviation; non-normally distributed variables as median (25^th^-75^th^ percentile). Categorical variables were presented as frequency (percentage). Comparison between groups were assessed by using student *t* test or one-way ANOVA for parametric data, Mann-Whitney *U* test or Kruskal-Wallis test for nonparametric data, and Pearson Chi-Square test for categorical data. Spearman correlation coefficient was used to analyze the association of collateral status with radiological and clinical variables. Independent factors for good outcome were evaluated using logistic regression analysis. Variables from univariate analyses at *p* < 0.1 were considered to represent explanatory variables and were evaluated together by multivariate analysis. All statistical analysis were performed using SPSS, Version 19.0 (IBM, Armonk, New York). A *p* value < 0.05 was considered statistically significant.

## Results

### Study population

**Fig 2:**
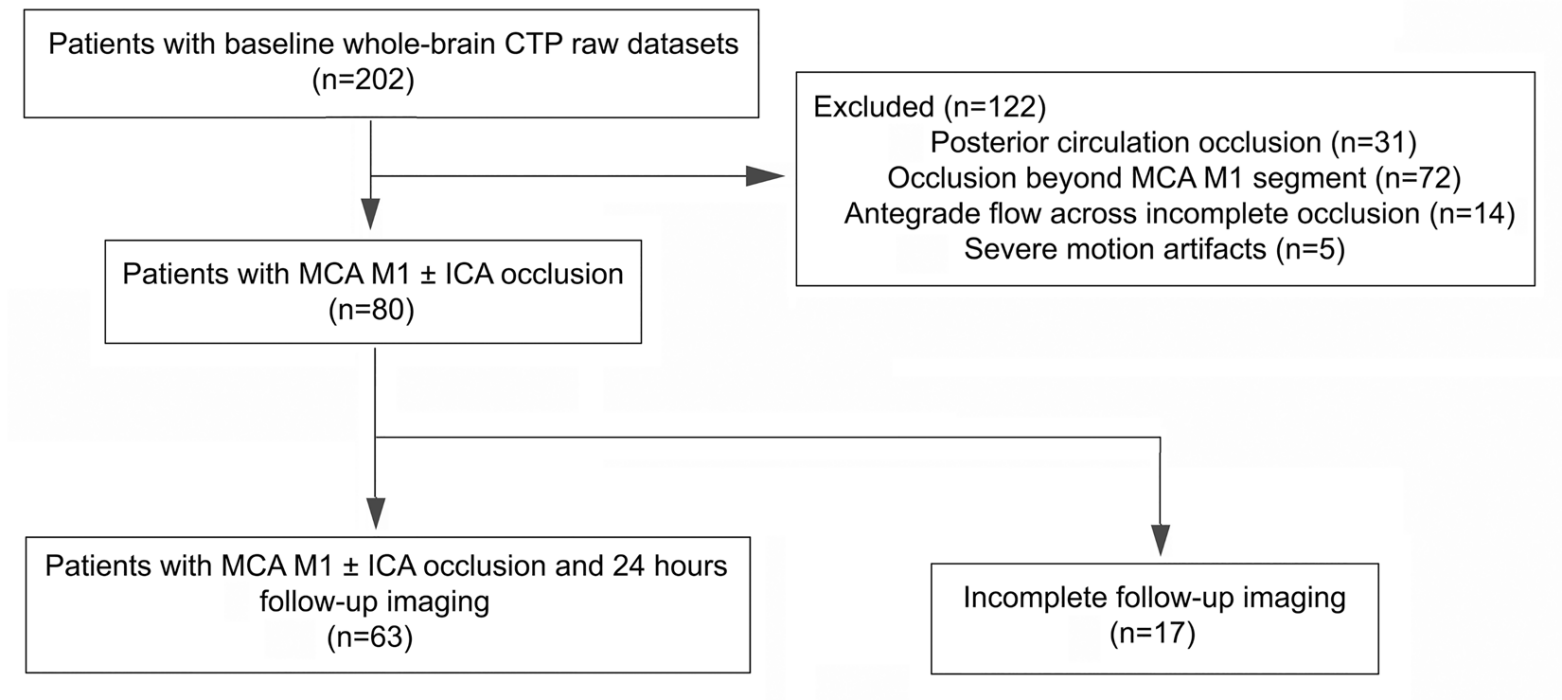
Inclusion and exclusion flow chart.

Among our cohort of 202 patients with whole-brain CTP raw datasets, 80 patients showed a MCA M1 segment and/or ICA occlusion on dynamic 4D CTA and were included for further analysis (Fig 2). The mean age of this population was 69 ± 6 years old with 33 (46.0%) being women. The median baseline NIHSS score was 13, and the median time from onset to start of CTP scanning and bolus of rt-PA was 169 minutes and 195 minutes, respectively. Additional ICA occlusion was presented in 23 patients (28.8%). All 80 patients received IVT and 13 (16.3%) underwent additional mechanical thrombectomy, 24 (30%) patients achieved good outcome. Of 13 patients received bridging thrombectomy, 12 (92.3%) patients achieved successful recanalization. The median time from onset to puncture and recanalization was 305 minutes and 418 minutes, respectively.

Of 80 patients, 63 had complete follow-up imaging and could therefore be assessed for recanalization, hemorrhagic transformation and final infarct volume (Table 1). 24-hours recanalization was observed in 36 (57.1%) patients, while only 16 (44.4%) of whom achieved good outcome. Follow-up CT was done in 36 patients (57.1%) and MRI in 27 patients (42.9%). Recanalization that identified from CTA and TOF-MRA were not significantly different.

**Table 1:** Baseline characteristics of patients.

	All patients (n = 80)	Subgroup with follow-up imaging (n = 63)
Age, y	69.2 ± 11.3	69.8 ± 11.1
Female, n (%)	33 (41.3)	29 (46.0)
Additional ICA occlusion, n (%)	23 (28.8)	20 (31.7)
OIT, min	169 (98–237)	175 (105–243)
ONT, min	195 (133–267)	202 (132–272)
Risk factors		
Hypertension, n (%)	54 (67.5)	44 (69.8)
Diabetes, n (%)	11 (13.8)	8 (12.7)
Atrial fibrillation, n (%)	48 (60.0)	38 (60.3)
hyperlipidemia, n (%)	35 (43.8)	30 (47.6)
Smoking, n (%)	20 (25.0)	14 (22.2)
History of stroke/ TIA, n (%)	10 (12.5)	7 (11.1)
Previous use of antiplatelet, n (%)	9 (11.3)	5 (7.9)
Baseline serum glucose, mg/mL	7.52 ± 3.13	7.83 ± 3.43
Baseline SBP, mm Hg	148.3 ± 21.7	148.6 ± 21.1
Baseline DBP, mm Hg	83.0 ± 12.9	83.2 ± 13.8
Baseline NIHSS	13 (10–17)	13 (10–17)
Baseline infarct volume, mL	64.2 (32.9–94.5)	65.7 (30.7–97.3)
Baseline hypoperfusion volume, mL	131.7 (90.5–203.7)	134.2 (89.8–217.2)
Baseline mismatch ratio	3.1 ± 2.8	3.2 ± 2.7

ICA: internal carotid artery; OIT: onset to imaging time; ONT: onset to needle time; TIA: transient ischemic attack; SBP: systolic blood pressure; DBP: diastolic blood pressure; NIHSS: national institute of health stroke scale.

### Association of rLMC of peak phase and tMIP with radiological and clinical outcome

There was a strong correlation between rLMC-P and rLMC-M scores (*ρ* = 0.835, *p* < 0.001) among all patients. In univariate analysis, rLMC-P (median: 11 *vs* 14, *p* < 0.001) and rLMC-M (median: 14 *vs* 17, *p* < 0.001) scores were significantly higher in patients with good outcome when compared to those with poor outcome (Table 2). In multivariate analysis, rLMC-P (OR, 1.372, 95% CI, 1.064–1.769, *p* = 0.015) and rLMC-M (OR, 1.353, 95% CI, 1.029–1.779, *p* = 0.030) were both independent predictors for good outcome after adjusting age, hyperlipidemia, additional ICA occlusion and baseline NIHSS score, with area under curve of 0.78 (95% CI, 0.677–0.888, *p* < 0.001) and 0.75 (95% CI, 0.643–0.859, *p* < 0.001), respectively.

**Table 2:** Univariate comparison for baseline clinical and imaging data.

	Poor outcome (mRS score, 3–6)	Good outcome (mRS score, 0–2)	*p* Value
	(n = 56)	(n = 24)	
Age, y	71.5 ± 11.1	63.9 ± 10.0	0.005
Female, %	23 (41.1)	10 (41.7)	0.960
Additional ICA occlusion, %	22 (39.3)	1 (4.2)	0.001
OIT, min	176.5 (110–237)	150.5 (86.8–245)	0.605
ONT, min	203.5 (135–271)	170 (129–252)	0.755
Risk factors			
Hypertension, n (%)	40 (71.4)	14 (58.3)	0.252
Diabetes, %	10 (17.9)	1 (4.2)	0.103
Atrial fibrillation, %	34 (60.7)	14 (58.3)	0.842
hyperlipidemia, %	28 (50.0)	7 (29.2)	0.085
Smoking, %	13 (23.2)	7 (29.2)	0.573
History of stroke/ TIA, %	7 (12.5)	3 (12.5)	1.000
Previous use of antiplatelet, %	5 (8.9)	4 (16.7)	0.315
baseline serum glucose, mg/mL	7.8 ± 3.6	6.8 ± 1.3	0.193
baseline SBP, mm Hg	148.3 ± 22.0	148.3 ± 21.5	0.994
baseline DBP, mm Hg	82.0 ± 12.5	85.3 ± 13.8	0.300
Baseline NIHSS	14 (12–18)	8.5 (5.25–12.0)	<0.001
Baseline infarct volume, mL	73.6 (36.9–111.1)	46.9(23.0–70.0)	0.001
Baseline hypoperfusion volume, mL	153.2 (100.7–215.4)	119.4 (75.8–146.2)	0.029
Baseline mismatch ratio, %	2.89 ± 2.38	3.61 ± 3.80	0.350
rLMC-P	11 (8.25–13)	14 (12.3–15.8)	<0.001
rLMC-M	14 (12–16)	17 (14–18)	<0.001

ICA: internal carotid artery; OIT: onset to imaging time; ONT: onset to needle time; TIA: transient ischemic attack; SBP: systolic blood pressure; DBP: diastolic blood pressure; NIHSS: national institute of health stroke scale

### Integrated collateral grading scale (CGS)

According to our novel CGS, 23 (28.8%) patients was scaled as good collaterals (CGS score of 2), 16 (20%) with intermediate (CGS score of 0) and 41 (51.3%) with poor collaterals (CGS score of 0). Patients with intermediate and good collaterals (CGS score of 1 and 2) had significantly lower baseline NIHSS score, smaller infarct and hypoperfusion volume, smaller final infarct volume when compared with poor collaterals (CGS score of 0) (all, *p* < 0.001). The rate of good outcome was higher in CGS score of 2 (65.2% vs 9.8%, OR, 10.349, 95% CI, 2.204–48.603, *p* = 0.003), compared with CGS score of 0 (Table 3). About 96.5% good outcome occurred in patients with intermediate and good collaterals, while only 51.3% or 62.5% was observed in patients with rapid (rLMC-P>11) or complete collaterals (rLMC-M>16). ROC analysis also showed CGS score had a larger area under curve (0.80, 95% CI, 0.689–0.912, *p* < 0.001) in predicting good outcome than either rLMC-P or rLMC-M.

**Table 3:** Clinical and imaging characteristics stratified by CGS score.

	Poor collaterals	Intermediate collaterals	Good collaterals	*p* Value
	(score of 0) (n = 41)	(score of 1) (n = 16)	(score of 2) (n = 23)	
Age, y	70.3 ± 11.5	71.1 ± 9.5	65.9 ± 11.8	0.241
Female, n (%)	14 (34.1)	8 (50.0)	11 (47.8)	0.413
OIT, min	176.4 ± 76.1	185.8 ± 98.0	167.1 ± 94.2	0.798
ONT, min	201.5±79.4	226.3±103.4	193.7±91.0	0.504
Baseline NIHSS score	15 (13–18.5)	12.5 (9–14)	10 (6–14)	<0.001
Baseline infarct volume, mL	92.8 (66.2–138.0)	39.6 (19.6–59.8)	37.5 (11.1–49.2)	<0.001
Baseline hypoperfusion volume, mL	192.9 (138.1–241.3)	98.6 (76.4–125.2)	100.6 (71.2–129.0)	<0.001
Baseline mismatch ratio	2.2 ± 1.2	3.6 ± 2.3	4.3 ± 4.5	0.017
Final infarct volume ^a^, mL	52.8 (22.6–166.6)	18.3 (9.5–44.1)	16.1 (2.0–48.0)	<0.001
Recanalization ^a^, n (%)	16(48.5)	9 (64.3)	11 (68.8)	0.156
sHT ^a^, n (%)	6 (18.2)	1(7.1)	0 (0)	<0.001
Good outcome, n (%)	4 (9.8)	5 (31.3)	15 (65.2)	<0.001
Death, n (%)	10 (24.4)	2 (12.5)	0 (0)	0.031

^a^ In patients with complete follow-up imaging alone (n = 63).

**Fig 3:**
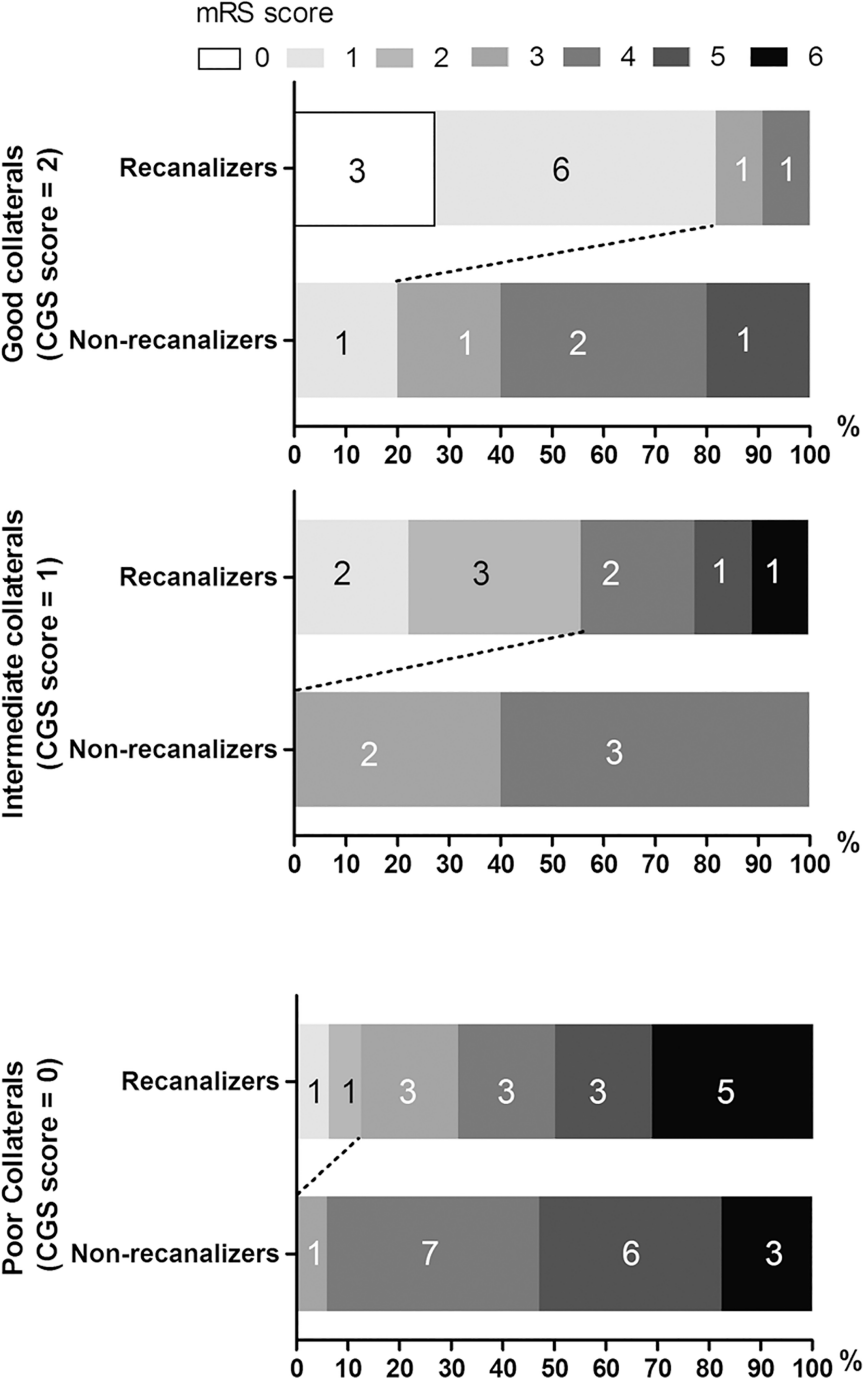
Clinical outcome measured on mRS score at day 90 stratified by CGS score and recanalization (AOL score ≥ 2).

In patients with complete follow-up imaging data, the interaction between CGS score and recanalization was relevant (*ρ* = 0.26, *p* = 0.039). However, regardless of the achievement of recanalization, Spearman correlation analysis demonstrated a significant decrease in 3-months mRS score across increasing CGS score (non-recanalizers: *ρ* = -0.495, *p* = 0.01; recanalizers: *ρ* = -0.671, *p* < 0.001). In patients with intermediate and good collaterals, rate of good outcome was significantly higher in patients who achieved recanalization than patients who did not (55.6% *vs* 0%, *p* = 0.038, 81.8% *vs* 20.0%, *p* = 0.018), while in patients with poor collaterals, this difference was not significant (12.5% *vs* 0%, *p* = 0.227) (Fig 3). Moreover, a higher rate of sHT was associated with a poorer collaterals in patients who achieved recanalization (*p* = 0.041). 85.7% (6/7) of sHT occurred in patients who had poor collaterals with subsequent recanalization, 83.3% (5/6) of whom were dead within 90 days after stroke, whereas no patient with poor collaterals had sHT when recanalization was not achieved.

## Discussion

In the current study, we firstly developed a collateral grading scale on 4D CTA, which integrated information of both velocity and extent of collaterals. Based on this scale, good collaterals were revealed to be independently associated with good outcome, especially when recanalization was achieved. However, patients with poor collaterals might not benefit from recanalization.

Our collateral grading scale provides a novel modality for comprehensive assessment of collateral status on dynamic 4D CTA. It has several strengths. Firstly, our CGS score was generated from 4D CTA which can mostly analogue collateral flow performed on DSA, and thus provides direct observation of the retrograde blood flow through collateral routes. Although some studies have used perfusion parameters to reflect collateral status, it has to be noticed that they still represent a perfusion characteristic related to the flow of the contrast bolus from the selected feeding artery to the respective tissue site. Furthermore, they are indirect markers which may be greatly influenced by cerebral venous circulation, for example, cerebral venous steal [15]. In cerebral venous steal, leptomeningeal collaterals may serve as pathways for venous flow diversion due to the increased inflow resistance and focal compression after infarction. In this case, the amount of collateral vessels may not be altered but tissue perfusion has been influenced. Therefore, grading collaterals on 4D CTA is better than that through CTP parameters. Secondly, our CGS integrates the advantages of rLMC-P and rLMC-M. The tMIP reconstruction that we used to assess the extent of LMCs was proved to visualize most complete vasculatures of collaterals because it recruited the late filling collaterals and averted fast washout of contrast medium on the unaffected side [8]. Moreover, we used peak phase as a single phase snapshot to identify the back-filling speed of LMCs, which can be independent of the different time resolution across centers. The collateral grading proposed by Menon et al was based on the scanning with 19 time points over 60 seconds, while the other CTP studies acquired images every second (total acquisition time, 44 seconds), which may make it difficult to be duplicated based on pure time in different centers [16,17]. Thirdly, peak phase and tMIP images can be generated directly using CTP raw data, additional scan time is not needed. Besides, the reconstruction of peak phase and tMIP are reproducible and fast, because dynamic 4D CTA clearly reveals each phase of contrast passage and the postprocessing by automatic software like MIStar is very simple. Therefore, this novel CGS may save the time on clinical decision based on collateral status and provide repeatable performances among different centers.

Based on our CGS, evidence was further reinforced that collateral status was associated with the neurological outcome after IVT. Patients with intermediate and good collaterals were most likely to benefit from recanalization, while patients with poor collaterals did not show a different response to recanalization. Moreover, our finding that a high rate of sHT and death occurred in patients with poor collaterals who were recanalized may support the concept of reperfusion injury. Reperfusion injury has been postulated to be a cause of severe hemorrhagic transformation in patients who achieved reperfusion [18]. Poor collaterals may limit effective reperfusion, even when recanalization is achieved. In contrary, sHT was observed in none of patients with good collaterals, probably because the degree of ischemic vascular injury can be minimized by abundant collateral, which made the hypoperfusion area reversible [19]. Thus identification of good collaterals based on our collateral scale may help to enhance medical decision-making and evaluate the cost effectiveness, especially in patients who can be quickly moved to the angiography suite.

The results of this study should be interpreted with caution because it is not a randomized controlled clinical trial. Validation and extension in larger and multi-center cohorts is needed. Second, this study might have a potential risk of selection bias that some severe stroke patients were not included in subgroup analysis because they might be transferred to intensive care unit or receive surgical treatment next day and thus have no follow-up images. Third, the use of both CT and MRI as follow-up imaging might result in heterogeneity of volume measurement. Moreover, follow-up TOF-MRA is not as objective as CTA to reveal vessel structure. However, it may not affect our results since the identified recanalization rate was comparable between two imaging. Finally, although strong evidence has validated the efficacy of bridging approach (IVT bridging with thrombectomy), the majority of our patients received IVT only. But our study focused on the impact of recanalization on the association between collateral status and outcome, which will also provide evidence for selecting eligible patients who will benefit from recanalization after endovascular therapy in future studies. Furthermore, it was more objective to reflect the prognosis value of collateral status by testing our collateral scores in IVT patients, because it avoided the influence from the disparity of surgical skills in different stroke centers to clinical outcome.

In conclusion, we firstly generated a novel collateral scale CGS based on 4D CTA, which proved to have prognostic value for clinical outcome in patients with reperfusion therapy. Patients with relative good collaterals were more likely to benefit from recanalization, whereas patients with poor collaterals showed no such response to recanalization but a high rate of sHT and death. Further validation of this collateral grading scale in patient selection for reperfusion therapy is still warranted in future larger cohorts.

## Supporting Information

S1 File Raw data of patients enrolled in this study. (XLS)

S2 File Raw data of new-adding patients received thrombectomy. (XLS)
